# Phase 2, open-label INSPIRE trial to assess the tolerability and effectiveness of transdermal cannabidiol gel in children and adolescents with 22q11.2 deletion syndrome (ZYN2-CL-031)

**DOI:** 10.1186/s11689-026-09687-z

**Published:** 2026-03-26

**Authors:** Helen Heussler, Jonathan Cohen, Caroline B. Buchanan, David S. Albers, Kristen G. Bzdek

**Affiliations:** 1 Centre for Clinical Trials in Rare Neurodevelopmental Disorders, Children’s Health Queensland Hospital and Health Services, South Brisbane, Queensland Australia; 2 Centre for Child Health Research, University of Queensland, South Brisbane, Queensland Australia; 3 Fragile X Alliance Inc., North Caulfield, Victoria Australia; 4 Victorian Clinical Genetic Services and Murdoch Children’s Research Institute, Melbourne, Victoria Australia; 5 Greenwood Genetic Center, Greenville, South Carolina USA; 6 Harmony Biosciences, Plymouth Meeting, Pennsylvania USA

**Keywords:** 22qDS, 22q11.2DS, 22q deletion syndrome, Anxiety, DiGeorge syndrome, Endocannabinoid system, Irritability, Safety, Transdermal cannabidiol

## Abstract

**Background:**

22q11.2 deletion syndrome (22qDS) is a genetic disorder affecting various body systems and associated with behavioral and psychiatric conditions, with no currently approved pharmacological treatments.

ZYN002, a transdermal gel containing synthetic cannabidiol, has been demonstrated to be well tolerated, showing potential benefit in related neurodevelopmental disorders. The INSPIRE study evaluated the safety, tolerability, and effectiveness of ZYN002 in children and adolescents with 22qDS.

**Methods:**

INSPIRE was a phase 2, open-label, multicenter trial comprising a 14-week treatment period (Period 1) followed by an optional 24-week, open-label extension (Period 2) for participants with ≥35% improvement on the Aberrant Behavior Checklist-Community (ABC-C) irritability subscale upon Period 1 completion.

Participants aged 4 to <18 years with genetically confirmed 22qDS, Clinical Global Impression-Severity score ≥4, and Pediatric Anxiety Rating Scale-Revised (PARS-R) score ≥10, both at Screening and Visit 2 (Day 1), were enrolled. Weight-based, transdermal ZYN002 administration occurred twice daily. Primary outcomes were safety and tolerability, including the incidence of treatment-emergent adverse events (TEAEs). Secondary endpoints included the PARS-R; Anxiety, Depression, and Mood Scale (ADAMS); ABC-C; Clinical Global Impression-Improvement (CGI-I), and Qualitative Caregiver-Reported Behavioral Problems survey.

**Results:**

Twenty participants initiated treatment; 17 completed Period 1, and 13 continued into Period 2. ZYN002 was well tolerated; only mild TEAEs were experienced in 35% of participants, and 15% reported treatment-related AEs. Upon Period 1 completion, significant changes from Baseline were observed across all measures: 40.6% PARS-R reduction (*P*=0.0005); 45.3% ADAMS total score reduction (*P*=0.0005) with clinically meaningful improvements in all subscales; and significant improvements in all ABC-C subscales, including irritability (36.3%, *P*=0.0055). On the CGI-I, 75% of participants were rated as “improved” or better relative to Baseline. For the behavioral problems survey, >80% of caregivers reported improvement in ≥1 problem during each period. Anxiety and irritability scores from Baseline through Period 2 maintained a similar reduction to Period 1.

**Conclusions:**

In this interventional, open-label trial of pediatric participants with 22qDS, ZYN002 was considered safe and well tolerated, and was associated with reductions in anxiety-related and behavioral symptoms, supporting further investigation of ZYN002 in a phase 3 randomized controlled trial.

**Trial registration:**

Clinicaltrials.gov (NCT05149898) registered on October 5, 2021.

## Introduction

### 22q11.2 deletion syndrome

22q11.2 deletion syndrome (22qDS) is one of the more common chromosomal disorders [1], with a prevalence of 1 in 3000 to 6000 live births [2, 3]. The most common deletion in 22qDS is a 3-Mb hemizygous deletion at chromosome 22q11.2, with a smaller nested deletion of 1.5-Mb in individuals with 22qDS who inherited the deletion from an affected parent [4]. The characteristic physical midline defects seen in 22qDS include congenital heart defects, cleft palate, distinctive facial features, and underdeveloped parathyroid glands that result in low calcium levels [2]. Additionally, up to 66% of children with 22qDS are at increased risk for several neurobehavioral and neuropsychiatric disorders, including symptoms of anxiety, social withdrawal, attention-deficit/hyperactivity disorder (ADHD), cognitive impairment, autism spectrum disorder (ASD), and mood disorders, as well as psychotic disorders and schizophrenia, which typically develop in early adolescence or early adulthood [5]. Notably, a constellation of anxiety and behavioral symptoms are similar across 22qDS, fragile X syndrome (FXS), and ASD [6].

There are currently no approved pharmacological treatments for 22qDS, including any that would directly address its neuropsychiatric, neurobehavioral, and neurodevelopmental manifestations. Management is typically supportive and focuses on treating the underlying medical conditions associated with the syndrome, such as congenital heart disease, hypocalcemia due to hypoparathyroidism, immune dysfunction, and palatal anomalies. Psychiatric and behavioral symptoms are managed with pharmacologic interventions as indicated, in combination with occupational, speech, and behavioral therapy and supportive educational services. However, these multidisciplinary approaches are largely aimed at alleviating symptoms, and treatments specific to this unique population continue to be pursued [3].

ZYN002, an investigational drug product in clinical development, is the first and only topically administered gel that is delivered transdermally and contains synthetic cannabidiol (sCBD) as the active ingredient. CBD is one of the > 140 different types of phytocannabinoids found in the *Cannabis sativa L.* plant and is the primary non-euphoric and non-psychoactive cannabinoid. CBD shows widespread biological effects in the central nervous system (CNS), including the endocannabinoid system (ECS) and beyond, and is thought to modulate brain signaling pathways by a multitude of mechanisms of action (MOAs), many of which remain unidentified to date [7]. ZYN002 has been evaluated in several indications including FXS and ASD.

### Cannabinoids in 22qDS

CBD, a non-intoxicating phytocannabinoid, has garnered increasing interest for its potential therapeutic effects across a range of neurodevelopmental, neurobehavioral, and neuropsychiatric disorders. Of note, the United States (US) Food and Drug Administration (FDA) has approved Epidiolex (cannabidiol), an oral, plant-derived form of CBD, for the treatment of seizures associated with some forms of developmental and epileptic encephalopathy [8–11]. CBD has demonstrated anxiolytic, antipsychotic, anti-inflammatory, and neuroprotective properties [12–15], and ZYN002 has shown effectiveness in behavioral symptoms with a favorable safety profile in pediatric populations [16–18], while tetrahydrocannabinol (THC) has been associated with an increase in psychotic symptoms [19]. ZYN002 does not contain THC and avoids the potential for conversion to THC in the acidic environment of the stomach and first pass hepatic metabolism due to its transdermal absorption [20]. Its putative MOAs, which include modulation of the ECS and other CNS neurotransmitter pathways, may be particularly relevant to the neuropsychiatric and other behavioral symptoms observed in 22qDS through CBD-specific pathways [5, 16–18, 21].

The chromosome deletions in 22qDS affect several genes that are essential for proper trafficking, endosomal recycling, and function in the ECS, including *CLTCL1* (clathrin heavy chain-like 1), *ZDHHC8* (zinc finger DHHC-type palmitoyltransferase 8), *SEPT5* (septin-5), and *SNAP29* (synaptosome-associated protein 29) [22–26]. The CB_1_ receptors in the CNS normally require these cellular processes for axonal trafficking and to respond to endogenous endocannabinoid signaling [27–29]. By virtue of its negative allosteric modulation of the endocannabinoid 2-arachidonoylglycerol (2-AG) [30, 31], CBD has the potential to normalize endocannabinoid signaling tone and β-arrestin-mediated desensitization of the CB_1_ receptor, reversing tachyphylaxis, and restoring endocannabinoid receptor function as previously described in FXS [17, 32].

Here we present the safety, tolerability, and effectiveness results of a phase 2, open-label study, ZYN2-CL-031 (INSPIRE: Assessing the impact of ZYN002 [transdermal CBD gel] on pediatric behavioral and emotional symptoms of 22qDS).

## Methods

### Study design

**Fig. 1:**
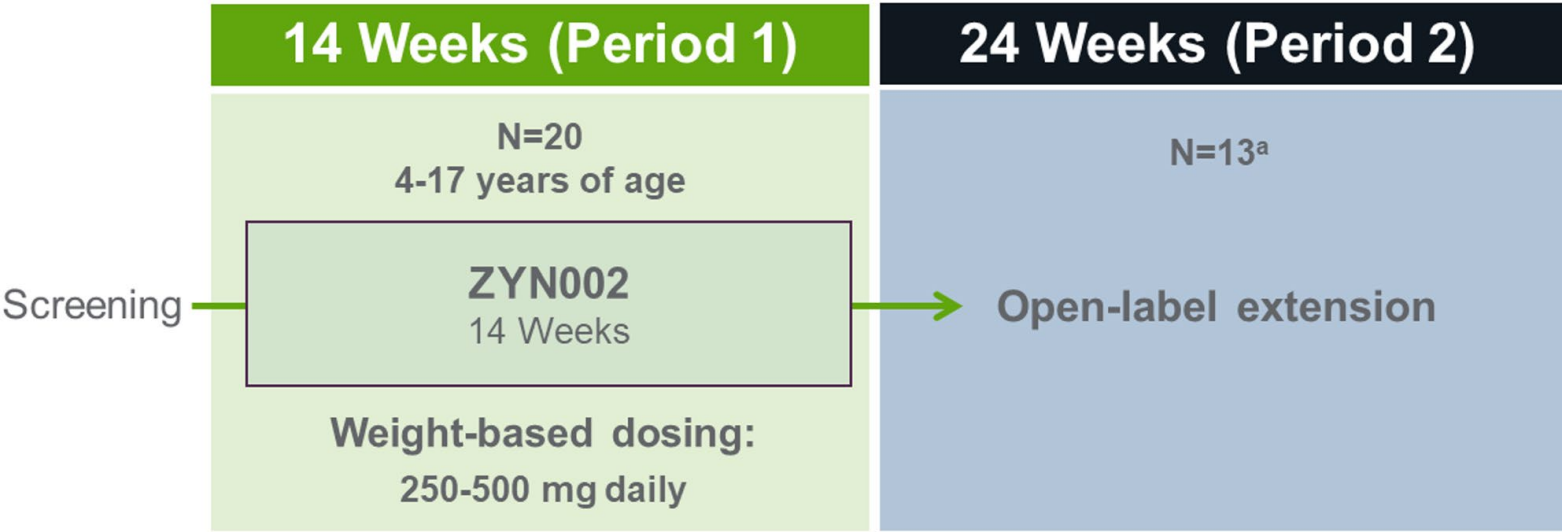
INSPIRE study design. ^a^Thirteen participants had a reported ≥ 35% improvement in the ABC-C irritability subscale score from baseline to Week 14 (end of Period 1) and were eligible and opted to continue on to Period 2. ABC-C, Aberrant Behavior Checklist-Community

INSPIRE was a phase 2, open-label, multisite trial conducted between February 2020 and November 2022 to evaluate the safety, tolerability, and effectiveness of ZYN002 sCBD gel (Harmony Biosciences). After Screening, participants began 14 weeks of treatment (Period 1), followed by an optional 24-week open-label extension (Period 2) for participants who demonstrated a ≥ 35% improvement from Baseline in the Aberrant Behavior Checklist-Community (ABC-C) irritability subscale at Week 14 (Fig. 1). This ≥ 35% improvement threshold was prespecified to reflect a robust change on a validated outcome measure widely used in studies of intellectual disability and ASD, distinguishing treatment-associated improvement from measurement variability or nonspecific effects, particularly in an open-label study. Response thresholds in the range of 25% to 40% have commonly been used in neurodevelopmental clinical trials, and the selected cutoff is consistent with prior literature supporting the reliability and responsiveness of this scale in pediatric neurodevelopmental populations [33]. The dose of ZYN002 was weight-based: participants ≤ 35 kg received 125 mg every 12 h (for a total daily dose of 250 mg), and participants > 35 kg received 250 mg every 12 h (for a total daily dose of 500 mg). ZYN002 transdermal sCBD gel was applied to clean, dry, intact skin of the upper arms/shoulders, and was administered as an add-on to standard-of-care medications.

### Participants

Participants were eligible for entry if they met the following key inclusion criteria: aged 4 to < 18 years with genetically confirmed 22qDS, Clinical Global Impression-Severity (CGI-S) of ≥ 4 at Screening and Visit 2 (Day 1), and Pediatric Anxiety Rating Scale-Revised (PARS-R) score of ≥ 10 at Screening and Visit 2 (Day 1).

Exclusion criteria included, but were not limited to, the following: (1) a positive drug screen for sympathomimetic amines (e.g., amphetamines [unless prescribed]), benzodiazepines, buprenorphine, cannabinoids, methadone, cocaine (metabolites), and opiates (excludes barbiturates used as anti-epileptic drugs [AEDs]), including ethanol; (2) use of the following AEDs: clobazam, phenobarbital, ethosuximide, felbamate, carbamazepine, phenytoin, or vigabatrin; (3) use of any strong inhibitor/inducer of CYP3A4 or sensitive substrate for CYP3A4; (4) on stable treatment for > 6 months of more than 2 psychoactive medications at Screening or throughout the study (with the exception of 1 psychoactive medication prescribed for sleep); (5) expected to initiate or change pharmacologic or non-pharmacologic interventions during the course of the study.

Information regarding concomitant medications was collected throughout the study. The exclusion criteria were designed to minimize potential confounding effects of behavioral pharmacotherapy by requiring stable treatment for at least 6 months of not more than 2 psychoactive medications at Screening and throughout the study. The planned sample size of 20 participants was determined based on feasibility considerations inherent to studying a rare neurodevelopmental disorder rather than on formal power calculations. Enrollment targets reflected realistic enrollment estimates across participating sites, which were initially limited to Australia, with 1 US site added subsequently. Study initiation and enrollment occurred during the coronavirus disease 2019 (COVID-19) pandemic, which further constrained recruitment.

### Outcome measures

The primary outcome measures were safety and tolerability, including the incidence of treatment-emergent adverse events (TEAEs), which were assessed throughout the study from Screening until study completion or early discontinuation. AEs were monitored continuously and assessed at each study visit, including clinic visits on Day 1, Week 6, and Week 14, during which outcome measures were collected. At each visit, the investigator monitored each participant for evidence of drug intolerance or the development of clinical and/or laboratory evidence of an AE.

There were several secondary outcome measures to assess effectiveness. First, the PARS-R is a clinician-administered instrument that measures parent/caregiver assessments of 61 anxiety-related symptoms [34]. The PARS-R is validated for children and adolescents up to age 17 with intellectual disability, and is well correlated with parent-reported and physician ratings of anxiety [35]. Using a 5-point Likert scale, the interviewer assesses 7 anxiety domains, including number, frequency, and severity of anxiety symptoms; severity of physical symptoms of anxiety; avoidance of anxiety-provoking situations; and interference in family/other relationships. For clinical trials, a total severity score is determined by summing 5 of the 7 domain scores. Higher scores reflect greater severity/impairment [34]. Second, the Anxiety, Depression, and Mood Scale (ADAMS) assesses behavior-based affective symptoms of individuals, consisting of 28 items that produce a total score and also 5 subscale scores, including hyperactive behavior, depressed mood, social avoidance, general anxiety, and obsessive-compulsive behavior [36]. Each item is scored from 0 (behavior has not occurred or is not a problem) to 3 (behavior occurs often or is a severe problem). Third, the ABC-C is an observer-reported and investigator-scored outcome measure assessing inappropriate and maladaptive behavior in individuals with intellectual and development disabilities across 5 behavioral domains: irritability, social withdrawal, stereotypic behavior, hyperactivity, and inappropriate speech [37, 38]. Fourth, the Clinical Global Impression-Improvement (CGI-I) was developed for clinicians in clinical trials to assess participants’ global functioning after intervention [39]. For the CGI-I, clinicians assessed how much the participant’s illness has improved or worsened relative to the Baseline/Severity state using two 7-point Likert-type scales, with lower scores reflecting greater improvements in symptoms. The CGI-I is well validated and correlates with other standardized measures of psychiatric severity [40–42]. Finally, for the Qualitative Caregiver-Reported Behavioral Problems survey, caregivers answered the following question at Baseline: *What are the 3 behavioral*,* emotional*,* or social problems that most impacted your son/daughter and his/her family in approximately the past year?* At subsequent visits they rated these self-identified behaviors as “improved,” “about the same,” or “worsened.”

Importantly, while advancement to Period 2 was primarily anchored to ≥ 35% improvement from Baseline in the ABC-C irritability subscale at Week 14, the protocol allowed for clinical discretion. The medical monitor could permit participation in Period 2 for participants demonstrating improvement in the ABC-C irritability subscale and the PARS-R, even if the participant had not fully reached 35% improvement on the ABC-C irritability subscale. This flexibility was intended to reduce the risk of excluding participants with meaningful multidomain clinical improvement.

### Statistical analysis

The population, demographics, baseline characteristics, and medical history were summarized using descriptive statistics, as was the incidence of TEAEs by system organ class and preferred term. Electrocardiogram (ECG) results and clinical laboratory tests were summarized using descriptive statistics, and shifts from Baseline to each visit were summarized descriptively. For physical/neurological examinations, shifts from Baseline to each visit were summarized using participant counts for each category. For all efficacy parameters, descriptive statistics for continuous data, and number and percentage for categorical data were used.

## Results

### Participant disposition

**Fig. 2:**
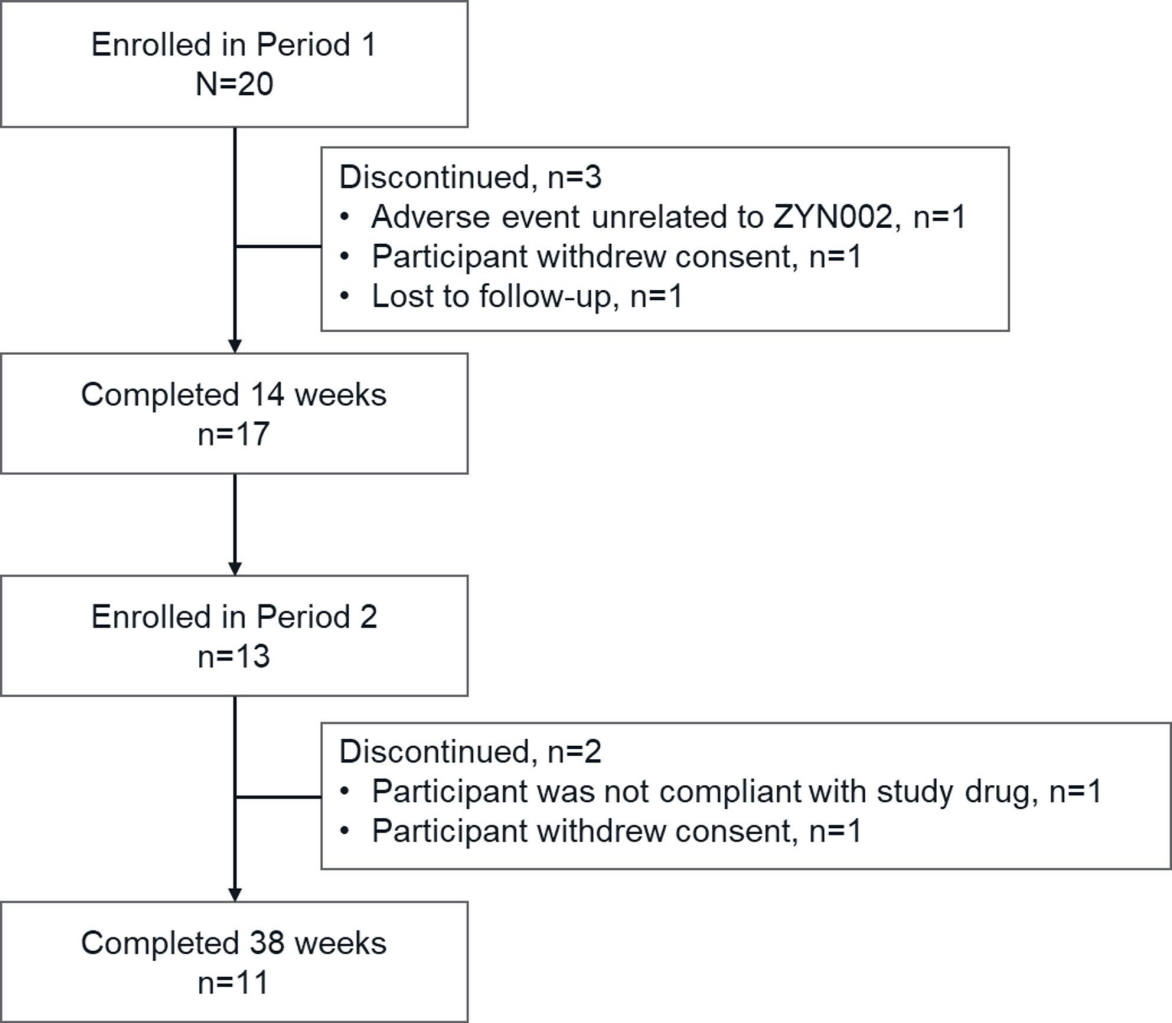
Participant disposition

After Screening, a total of 20 participants (aged 4–17 years at the time of entry) enrolled, and 17 participants completed the 14 weeks of Period 1 (Fig. 2). Thirteen participants had a reported ≥ 35% improvement in the ABC-C irritability subscale score at Week 14 relative to their respective baseline values and continued to Period 2. Notably, 1 participant was excluded from the final analysis due to protocol deviations and therefore could not enroll in Period 2. Twelve of the 13 participants who entered Period 2 completed 38 weeks of treatment, although 1 participant withdrew consent and another was not compliant with study drug; therefore, 11 participants were included in the analyses for Period 2.

### Baseline characteristics

Baseline demographics were collected for all participants in Period 1 (Table 1). Mean age (range) was 9.9 [4–15] years; the majority of participants were male (60.0%) and White (90.0%). Participant medical histories were reported at Baseline, including psychiatric, nervous system, congenital, and ear and labyrinth disorders (Table 1). Concomitant medications remained stable throughout the study in accordance with protocol requirements. Although 4/20 (20%) enrolled participants were reported as having a diagnosis of ASD based on their medical history at Baseline, Autism Diagnostic Observation Schedule-2 (ADOS-2) assessments at Screening indicated symptomatology indicative of ASD in 8/15 (53%) participants. It is unclear whether or to what extent ASD correlates to anxiety symptoms and/or language challenges. Two participants did not have an ADOS-2 evaluation because they were hearing impaired, and 3 participants were granted waivers because no psychologist was available to administer the assessment due to COVID-19 travel restrictions.

**Table 1:** Baseline characteristics and disorders in medical history

Baseline characteristics	Period 1
N	20
Mean age, years (range)	9.9 (4–15)
Sex, n (%)	
Male	12 (60.0)
Female	8 (40.0)
Race, n (%)	
White	18 (90.0)
Black or African American	1 (5.0)
Multiple	1 (5.0)
Median weight, kg	33.5
Range (min, max)	13.7, 79.8
Median BMI, kg/m^2^	17.9
Range (min, max)	13.4, 32.4
Disorder on medical history, n (%)	Period 1
Congenital disorders (e.g., aberrant aortic arch, cleft palate)	9 (45)
Ear and labyrinth disorder (e.g., conductive deafness)	8 (40)
Nervous system	12 (60)
Apraxia	2 (10)
Dyspraxia	1 (5)
Hypotonia	2 (10)
Intellectual disability	2 (10)
Language disorder	1 (5)
Mental impairment	3 (15)
Sensory processing disorder	3 (15)
Speech disorder development	4 (20)
Psychiatric	16 (80)
Anxiety	6 (30)
ADHD	6 (30)
ASD (by history)	4 (20)
Behavior disorder	2 (10)
Insomnia	5 (25)
Learning disability	2 (10)
Obsessive-compulsive disorder	1 (5)
Separation anxiety disorder	1 (5)
Sleep disorder	5 (25)

*ADHD *Attention-deficit/hyperactivity disorder, *ASD *Autism spectrum disorder, *BMI *Body mass index

### Adverse events and primary outcomes

During Period 1, 14 TEAEs occurred in 7 participants (35%), all of which were mild (Table 2).

No serious TEAEs were reported. There were 3 treatment-related AEs in 3 participants, including application site pain (2 participants) and application site pruritus (1 participant). One participant discontinued due to non-serious AEs of contusion, ADHD, and ASD, all of which were mild in severity and not related to study treatment. There were no clinically significant changes in laboratory results, ECG findings, or vital signs.

**Table 2:** Adverse events and safety in Period 1 (14 Weeks)

Adverse Event Type	Participants (*N* = 20) % Participants
TEAEs	35% (7 participants; 13 events)
Mild in severity	100%
Treatment-related AEs	15% (3 participants; 3 events)
Application site pain Application site itching	10% (2 participants; 2 events) 5% (1 participant; 1 event)
Discontinuations due to TEAEs unrelated to ZYN002	5% (1 participant)

*AE* Adverse event, *TEAE *Treatment-emergent AE

### Effectiveness outcomes

**Fig. 3:**
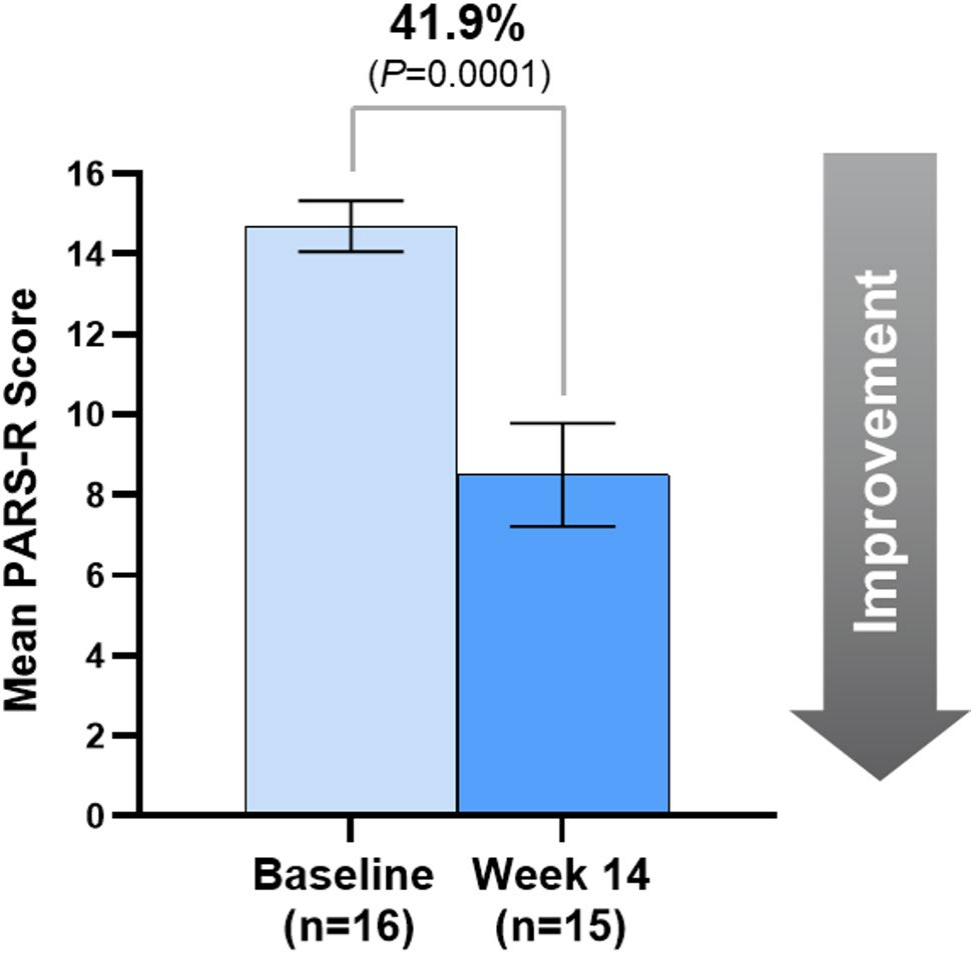
Significant improvement in mean pediatric anxiety rating scale-revised score between baseline and Week 14. Percentage above graph indicates percent difference between Baseline and end of Period 1/Week 14 score measurements. Bars represent mean ± standard error. The analysis included 16 participants at Baseline and 15 participants at Week 14 for PARS-R (1 participant did not have a valid assessment at Week 14 and 1 was excluded from the final analysis). PARS-R, Pediatric Anxiety Rating Scale-Revised

At Baseline, the mean±standard error (SE) PARS-R score (*n* = 16) was 14.7 ± 0.6 which indicates clinically meaningful moderate anxiety symptom levels. At the end of Period 1, the mean ± SE PARS-R score (*n* = 15) was 8.5 ± 1.3 which is consistent with mild anxiety symptom levels (Fig. 3). The 41.9% difference in PARS-R score from Baseline to Week 14 was statistically significant (*P *= 0.0001).

**Fig. 4:**
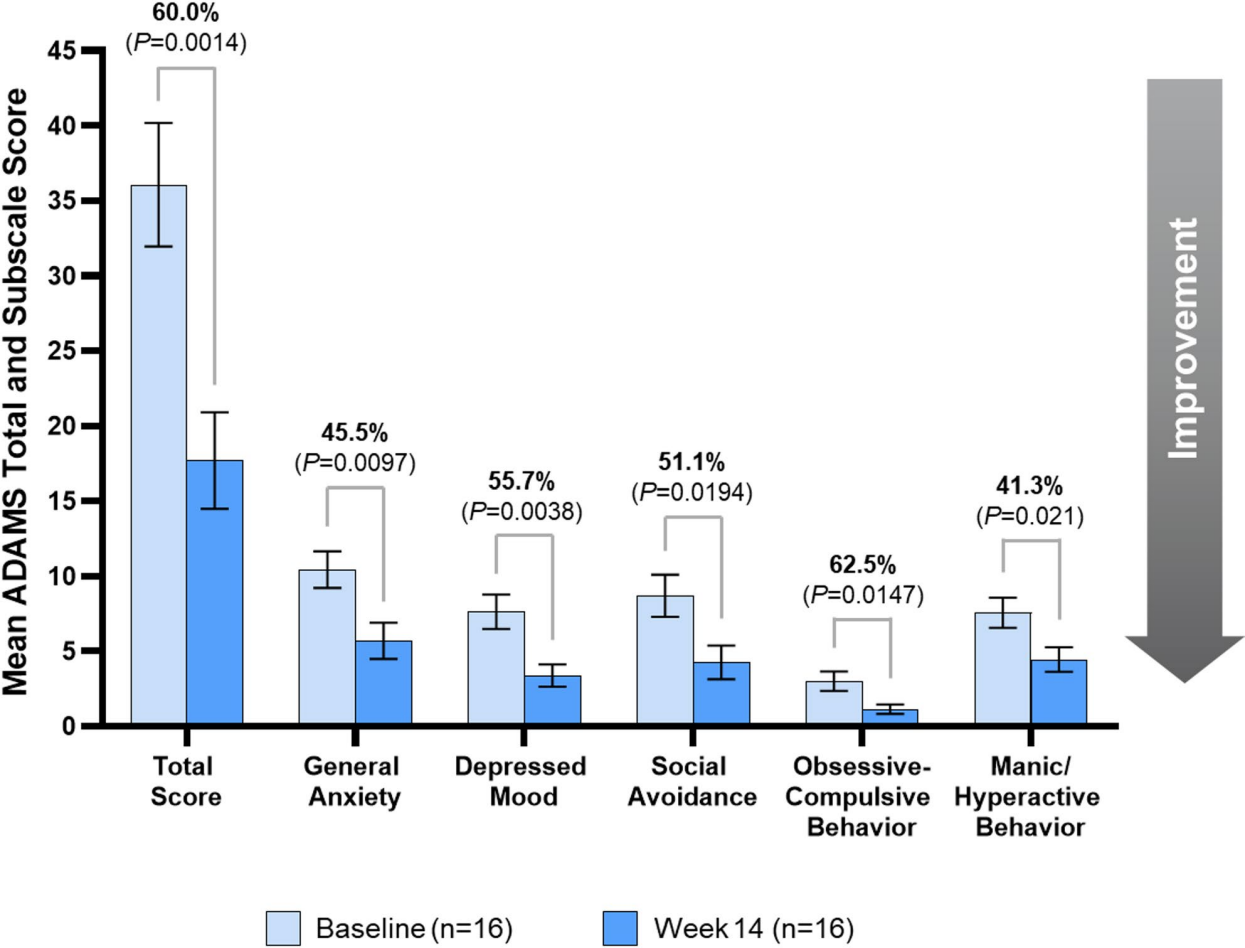
Significant improvement in mean anxiety, depression, and mood total and subscale scores between baseline and Week 14. Percentages above graphs indicate percent difference between Baseline and end of Period 1/Week 14 score measurements. Bars represent mean ± standard error. The analysis included 16 participants for ADAMS (1 participant was excluded from the final analysis). ADAMS, Anxiety, Depression, and Mood Scale

Behavioral symptoms were assessed using the mean ADAMS total and subscale scores (*n* = 16), which indicated clinically meaningful and statistically significant improvements from Baseline to Week 14 in the general anxiety subscore (45.5%, *P* = 0.0097), depressed mood (55.7%, *P* = 0.0038), social avoidance (51.1%, *P* = 0.0194), obsessive-compulsive behavior (62.5%, *P* = 0.0147), and manic/hyperactive behavior (41.3%, *P* = 0.021), as well as total score (60.0%, *P* = 0.0014) (Fig. 4).

**Fig. 5:**
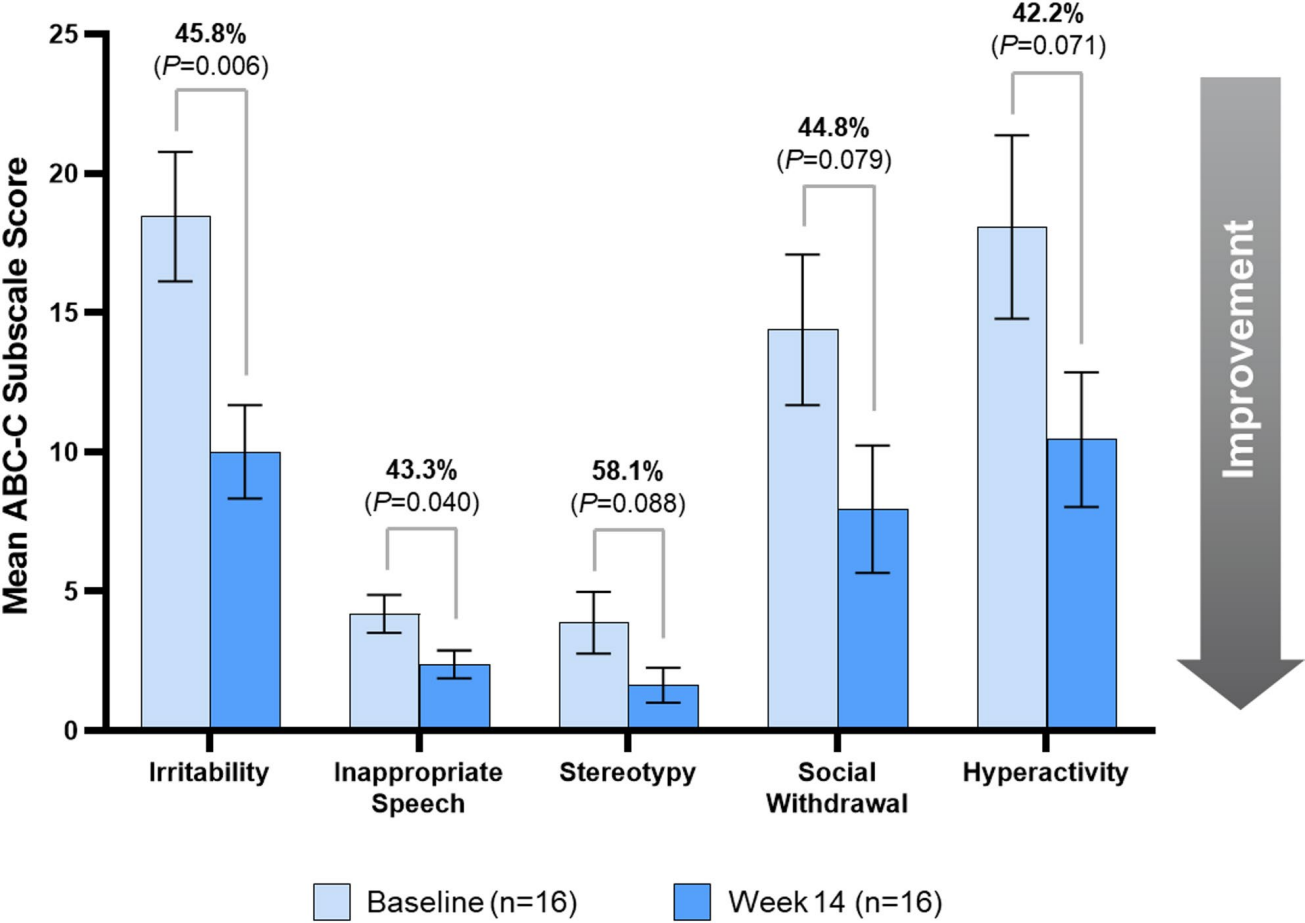
Significant improvement in certain aberrant behavior checklist-community subscale scores between baseline and Week 14. Percentages above graphs indicate percent difference between Baseline and end of Period 1/Week 14 score measurements. Bars represent mean ± standard error. The analysis included 16 participants for ABC-C (1 participant was excluded from the final analysis). ABC-C, Aberrant Behavior Checklist-Community

Aberrant behaviors were assessed using the mean ABC-C subscale scores (*n* = 16), which indicated statistically significant improvements from Baseline to Week 14 in irritability (45.8%, *P* = 0.006) and inappropriate speech (43.3%, *P* = 0.040). Stereotypy (58.1%, *P* = 0.088), social withdrawal (44.8%, *P* = 0.079), and hyperactivity (42.2%, *P* = 0.071) showed numerical declines from Baseline to Week 14, but these changes did not indicate statistically significant improvements (Fig. 5). Clinicians rated participants (*n* = 16) at Week 14 using the CGI-I and noted clinically meaningful improvements. Clinicians rated 75% of participants as “improved,” “much improved,” or “very much improved,” including 62.5% of participants who were rated as “much improved” or “very much improved.” For the Qualitative Caregiver-Reported Behavioral Problems survey, 82% of caregivers in Period 1 reported ≥ 1 problem improved. Among the 110 total codes used for the Qualitative Caregiver-Reported Behavioral Problems survey: 32% were within naturalistic, 31% irritability, 21% hyperactivity/noncompliance, 15% social withdrawal, 1% inappropriate speech, and 0% stereotypy. The naturalistic domain encompassed the majority of codes used throughout this study. Specifically, the code “anxiety” contained 18% of the responses given. Naturalistic codes were supplemented where apposite ABC-C codes were not applicable.

**Fig. 6:**
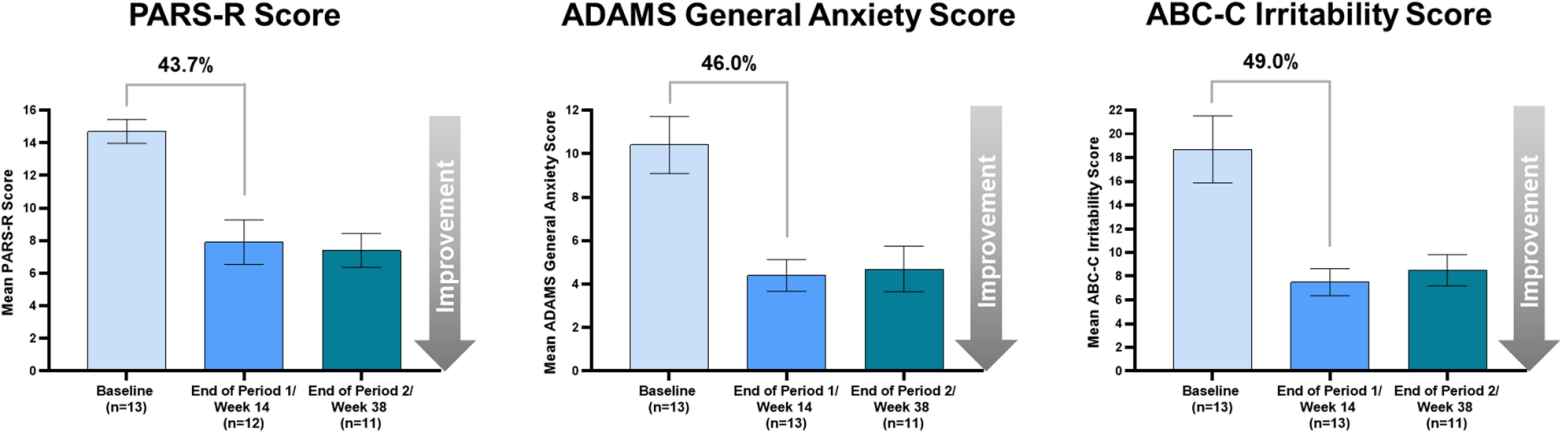
Sustained reduction in anxiety and irritability scores from baseline through Period 2 in participants who continued to Period 2. Percentages above graphs indicate percent difference between Baseline and end of Period 1/Week 14 score measurements. Bars represent mean ± standard error. Twelve of the 13 participants who entered Period 2 completed 38 weeks of treatment, although 1 participant withdrew consent and 1 participant was not compliant with study drug; therefore, 11 participants were included in the analyses for Period 2. ABC-C, Aberrant Behavior Checklist-Community; ADAMS, Anxiety, Depression, and Mood Scale; PARS-R, Pediatric Anxiety Rating Scale-Revised

For the 13 participants who had a reported ≥ 35% improvement from Baseline in the ABC-C irritability subscale score at Week 14 (end of Period 1) and continued to Period 2 (24-week duration, 11 participants completed/were included in Period 2), anxiety and irritability scores from Baseline through Week 38 (end of Period 2) maintained a similar reduction to Period 1. Additionally, for the Qualitative Caregiver-Reported Behavioral Problems survey, 85% of the caregivers for the participants who continued to Period 2 reported an improvement in ≥ 1 problem (Fig. 6).

## Discussion

There are currently no US FDA- or internationally approved medications for treating anxiety or other behavioral symptoms in children with 22qDS. In this phase 2, open-label INSPIRE study, the safety, tolerability, and effectiveness of ZYN002 were investigated in children and adolescents with 22qDS. ZYN002 was well tolerated and had a safety profile consistent with transdermal ZYN002 administration in other clinical trials [16–18]; no new safety signals emerged. At the end of Period 1 (i.e., Week 14), behavioral outcomes showed statistically significant improvements from Baseline in the PARS-R, the total score and all 5 subscales of the ADAMS, and in 2 subscales of the ABC-C. Additionally, there were clinically meaningful improvements in all 5 subscales of the ADAMS [36, 43] and in the CGI-I ratings. The alignment of improvements across multiple outcome measures reinforces the overall consistency and potential clinical significance of the observed symptom improvements.

Participants with a reported ≥ 35% improvement in the ABC-C irritability subscale score at Week 14 relative to their respective Baseline values were given the option to continue on to Period 2; the reduction in anxiety and irritability scores reported at the end of Period 1 were maintained through the end of Period 2. In addition to similar safety and tolerability profiles as previous studies, there was a statistically significant difference in response rate from Baseline to Week 14 for the ABC-C irritability and inappropriate speech subscale scores in this trial, which was consistent with a previous trial of ZYN002-treated participants with ASD [16]. Additionally, the CGI-I rated the majority of participants as improved in the current study, which is also consistent with a previous trials of ZYN002-treated participants with ASD and for a subpopulation of ZYN002-treated participants with FXS with ≥ 90% methylation of the promoter region of the *FMR1* (fragile X messenger ribonucleoprotein 1) gene [16–18].

Children and adolescents with 22qDS frequently exhibit elevated rates of anxiety disorders and social withdrawal, which are among the earliest and most prominent behavioral manifestations in this population. Approximately 40% of individuals with 22qDS suffer from some type of anxiety disorder [21], with phobias being the most common, followed by generalized anxiety disorder, separation anxiety, and obsessive-compulsive disorder [44]. These symptoms can emerge early in life and are shaped by various factors, including early negative experiences and serious medical complications such as congenital heart disease, feeding difficulties, and repeated surgeries or hospitalizations, all of which can disrupt the development of the physiological stress response and contribute to a poor sense of bodily control [45, 46]. Anxiety symptoms in this population may precede or co-occur with the emergence of mood disorders, attentional difficulties, and, in some cases, psychosis in adolescence or young adulthood; thus, highlighting anxiety symptoms as a key therapeutic target in efforts to improve long-term neuropsychiatric outcomes [47, 48]. Specifically, evidence suggests that anxiety and early-life trauma are significant risk factors for future development of psychosis [49, 50]. A robust body of longitudinal and cross-sectional research has consistently identified anxiety symptoms and social withdrawal as core features underlying the 22qDS neuropsychiatric phenotype, and as possible modifiable risk factors for later psychotic illness [51–55].

Given this potential developmental trajectory, early interventions that effectively reduce anxiety and related symptoms may have important implications for altering the course of psychiatric risk in those with 22qDS. While the INSPIRE trial focused on anxiety and other behavioral symptoms in a pediatric cohort that was mostly below the typical age of psychosis onset (but may have experienced pre-psychotic symptoms), reducing early symptoms of pre-psychosis with effective therapies may impact the long-term risk of development of psychotic disorders and schizophrenia in this high-risk population. Unlike THC, which is associated with exacerbation of psychosis and increased risk for the development of schizophrenia, including in individuals with 22qDS, CBD has demonstrated antipsychotic properties [13, 19, 56, 57]. ZYN002, which was found in a phase 1 study to contain no detectable THC in plasma or urine [58], may therefore provide a way to harness the potential antipsychotic effects of CBD without THC exposure, although further study in individuals with 22qDS is needed.

Disruption of the ECS is one of the proposed mechanisms underlying behavioral symptoms in children with neurodevelopmental disorders [59, 60], as shown in preclinical and clinical studies in children and adolescents with FXS. In FXS studies, transdermal treatment with ZYN002 was associated with clinically meaningful reductions in anxiety symptoms, social avoidance, and irritability [17, 18]. The ECS includes the CBD receptors, CB_1_ and CB_2_, which are 2 types of G-protein–coupled receptors [61, 62]. CBD acts as a negative allosteric modulator at presynaptic CB_1_ receptors [30]. Brain regions that possess high levels of CB_1_ receptors include the neocortex, cerebellum, and forebrain structures, as well as the basal ganglia and limbic system areas that contribute to learning and memory, executive functioning, social interaction, behavior, and emotion [62]. CB_2_ receptors are expressed primarily in the immune and hematopoietic systems, but they are also present in the brain, pancreas, and bone. The primary endogenous ligands for CB_1_ and CB_2_ receptors include anandamide and 2-AG which modulate synaptic transmission throughout the CNS, yielding widespread influence on cognition and behavior [63, 64]. In addition to the CBD receptors, CB_1_ and CB_2_ [30], CBD has shown activity at the serotonin 1 A (5HT_1A_) [65], gamma-aminobutyric acid type A [66], and D_2_ and D_3_ (dopamine) [67, 68] receptors. The multifaceted pharmacology of CBD provides support for its potential use in treating the behavioral symptoms associated with 22qDS, warranting further study in this condition.

CBD also inhibits the enzyme fatty acid amide hydrolase, which increases levels of anandamide. Elevated anandamide signaling has been linked to fewer psychotic symptoms and delayed onset of psychosis [13]. Specifically, a clinical study in schizophrenia found that CBD treatment significantly raised serum anandamide levels, which was significantly associated with symptom improvement, suggesting that inhibition of anandamide deactivation may contribute to the antipsychotic effects of CBD [13]. This mechanism is particularly relevant to the anxiety symptoms commonly seen in 22qDS, which may reflect the underlying ECS dysregulation. In the current study, improvements across validated behavioral scales may be attributable, in part, to enhanced anandamide signaling and ECS modulation. This is consistent with emerging models of affective dysregulation in neurodevelopmental disorders, in which anxiety symptoms are driven by dysfunction in neurotransmitter systems, including the endocannabinoid and serotonergic circuits. Notably, recent evidence by Alvino et al. highlights synaptic dysregulation and large-scale functional brain network disruptions during development in individuals with 22qDS. This includes a transition from hyper- to hypoconnectivity in key brain areas, which has been linked to glycogen synthase kinase 3β-mediated synaptic mechanisms and social deficits [55]. These insights further underscore the need to investigate how pharmacologic modulation of ECS components may interact with these developmental connectivity trajectories.

While the phase 3 RECONNECT study in participants with FXS demonstrated a notable response in those treated with ZYN002, the placebo group response was also substantial, limiting the ability to detect a statistically significant treatment effect [69]. Differences in clinical outcomes between the present study and RECONNECT may reflect important biological and phenotypic distinctions between 22qDS and FXS, i.e., the proposed mechanisms of action in these conditions involve different neurotransmitter pathways and different symptomatology, which may influence treatment responsiveness.

### Study limitations

The current study had an open-label design, no placebo arm, and a small participant population, which should be considered when interpreting the findings. Additionally, use of fixed, weight-based dosing may have led to more variability compared to dosing on an mg/kg basis, especially in higher-weight individuals. Subsequent studies of ZYN002 have allowed for more dosing ranges based on weight. Further, the study population was predominantly White due to the recruitment areas of this preliminary study, which may limit the generalizability of the findings to racially and ethnically diverse populations with 22qDS. Finally, although the PARS-R has been validated in developmentally disabled individuals and is administered through caregiver interview and incorporates observational reporting rather than relying solely on patient self-report, certain items may be more difficult to assess in minimally verbal individuals. As a result, variability in caregiver interpretation of these items may have influenced scoring in some participants.

## Conclusions

The results from the INSPIRE trial provide initial evidence showing that ZYN002 is safe and generally well tolerated in children and adolescents with 22qDS when added to a stable standard of care. The findings from this phase 2, open-label study encourage further investigation of ZYN002 in a phase 3 trial to assess safety and efficacy in this underserved participant population.

## Data Availability

All data generated or analyzed during this study are included in this published article.

## Abbreviations

2-AG: Endocannabinoid 2-arachidonoylglycerol
22qDS: 22q11.2 deletion syndrome
5HT_1A_: Serotonin 1A
ABC-C: Aberrant Behavior Checklist-Community
ADAMS: Anxiety, Depression, and Mood Scale
ADHD: Attention-deficit/hyperactivity disorder
ADOS-2: Autism Diagnostic Observation Schedule-2
AE: Adverse event
ASD: Autism spectrum disorder
BMI: Body mass index
CB_(1)(2)_: Cannabidiol receptors 1 and 2
CBD: Cannabidiol
CGI-I: Clinical Global Impression-Improvement
CGI-S: Clinical Global Impression-Severity
CLTCL1: Clathrin heavy chain-like 1
CNS: Central nervous system
COVID-19: Coronavirus disease 2019
D_(2)(3)_: Dopamine receptors 2 and 3
ECG: Electrocardiogram
ECS: Endocannabinoid system
FDA: Food and Drug Administration
FMR1: Fragile X messenger ribonucleoprotein 1
FXS: Fragile X syndrome
INSPIRE: Assessing the impact of ZYN002 (transdermal CBD gel) on pediatric behavioral and emotional symptoms of 22qDS
IRB: Institutional Review Board
MOA: Mechanism of action
PARS-R: Pediatric Anxiety Rating Scale-Revised
sCBD: Synthetic cannabidiol
SEPT5: Septin-5
SNAP29: Synaptosome-associated protein 29
TEAE: Treatment-emergent adverse event
THC: Tetrahydrocannabinol
US: United States
*ZDHHC8*: Zinc finger DHHC-type palmitoyltransferase 8
